# CPK12 and Ca^2+^-mediated hypoxia signaling

**DOI:** 10.1080/15592324.2023.2273593

**Published:** 2023-10-24

**Authors:** Santosh Kumar Upadhyay

**Affiliations:** Department of Botany, Panjab University, Chandigarh, India

**Keywords:** Calcium, CPK12, ERF-VII, hypoxia, signaling

## Abstract

Hypoxia triggers reactive oxygen species (ROS)-induced elevation in cytoplasmic calcium (Ca^2+^) in the plant cells. Calcium-dependent protein kinase 12 (CPK12) acts as a sensor to recognize the Ca^2+^ signature and is activated by autophosphorylation. Then, the CPK12 moves into the nucleus with the help of phosphatidic acid (PA) and phosphorylates ERF-VII family proteins that activate hypoxia signaling and response. The study provides a novel mechanism of hypoxia signaling in plants. Moreover, the mechanism of hypoxia-specific Ca^2+^ signature generation remains elusive.

## Introduction

Hypoxia in plants is usually created by water logging in roots, submergence or high O_2_ demand than the accessibility under certain tissues.^1^ It may also be caused by necrotrophic infections.^2^ Hypoxia results in less ATP synthesis due to a compromised respiration process, reduced supply of carbohydrates, and higher accumulation of harmful metabolites, which severely affect the growth and development of plants.^1^ Plants have also evolved numerous mechanisms including morphological, physiological and biochemical changes to reduce the impact of hypoxia.^3,4^

**Figure 1.**
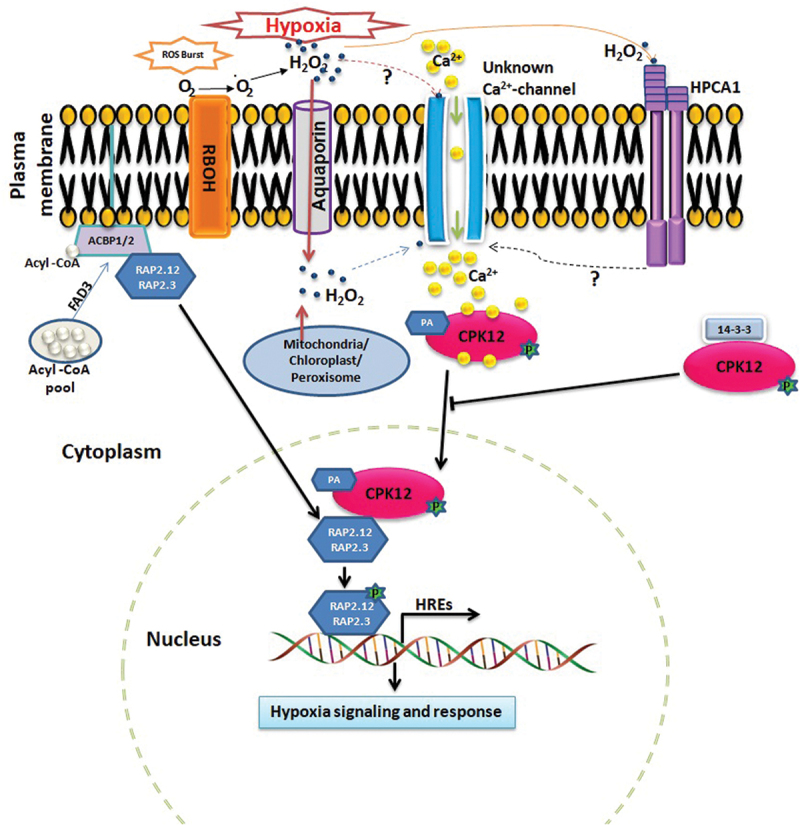
A model displaying the CPK12 as a central player in hypoxia signaling. Wu *et al*.^7^ described the hypoxia-induced accumulation of H_2_O_2_, which triggers reactive oxygen species (ROS)-induced Ca^2+^ influx in the cytoplasm by an unknown Ca^2+^channel. Moreover, how H_2_O_2_ activates the Ca^2+^ channel is elusive. Further, Wu *et al*.^7^ did not report the source of H_2_O_2._ it is not evident whether hypoxia-induced H_2_O_2_ is produced intracellularly in the organelles or extracellularly in the apoplast, and influx into the cytoplasm through a specific aquaporin channel as reported by Fichmann *et al*.^11^ In addition, Wu *et al*.^12^ reported hydrogen-peroxide-induced Ca^2+^ increases 1 (HPCA1)-mediated activation of the Ca^2+^ channel during various stress conditions. HPCA1 is a leucine-rich-repeat receptor-like kinase that acts as an extracellular H_2_O_2_ sensor and promotes systemic ROS and Ca^2+^ signaling.^11,12^ However, its role in Ca^2+^ influx during hypoxia is not studied. Fan *et al*.^10^ elaborated that the CPK12 acts as a sensor for hypoxia-induced Ca^2+^ signature in the cytoplasm, activates by autophosphorylation, and then translocates into the nucleus with the help of phosphatidic acid (PA). Inside the nucleus, CPK12 phosphorylates and stabilizes RAP2.12 and RAP2.3 transcription factors of the ERF-VII family that induce the expression of hypoxia-responsive elements (HREs) to start hypoxia signaling. Contrarily, the interaction of 14-3-3 protein prevents nuclear translocation of CPK12. Further, acyl-CoA binding proteins (ACBP1/2) and fatty acid desaturase (FAD3) facilitate the nuclear translocation of ERF-VII proteins from the plasma membrane through unsaturated acyl-CoA.^3^ The figure is modified from the study of Fan *et al*.^10^ with permission through RightsLink (License Number 5632361196904).

Calcium (Ca^2+^) signaling plays a vital role in numerous biological processes in plants. It starts with a condition-specific Ca^2+^ signature, which is decoded by a diverse range of sensor proteins in the cytoplasm to further transmit the signal for situation-specific downstream processes.^5,6^ An increase in cytoplasmic Ca^2+^ concentration during hypoxia is reported,^7^ which might act as a Ca^2+^ signature. The interaction of a sensor protein calcineurin B-like proteins 4 (CBL4) with CBL-interacting protein kinases 15 (CIPK15) triggers *α-amylase* genes through sucrose non-fermenting-1-related protein kinase-1 (SnRK1), and promotes growth in rice plants during hypoxia.^8^ In addition, *cml38* (a *calmodulin-like* gene) knockout mutants display enhanced sensitivity to hypoxia.^9^ Further, the hypoxia induces an increase in H_2_O_2_ concentration that triggers reactive oxygen species (ROS)-induced influx of Ca^2+^ in the cytoplasm.^7^ These reports suggest an interactive role of Ca^2+^ and ROS during hypoxia response in plants. However, which specific Ca^2+^ channel is precisely involved in the Ca^2+^ influx during hypoxia, and the mechanism of hypoxia-specific Ca^2+^ signature decoding in the cytoplasm is not known. Recently, Fan et al.^10^ revealed calcium-dependent protein kinase 12 (CDPK/CPK12) as a sensor protein for decoding the hypoxia-mediated Ca^2+^ signature that activates the downstream signaling through the phosphorylation cascade (Figure 1).

## Ca^2+^ signature and CPK12 phosphorylation during hypoxia signaling

Previous studies suggested an increase in Ca^2+^ concentration during hypoxia in *Arabidopsis*.^7^ The study by Fan *et al*.^10^ revealed the mechanism of CPK-mediated Ca^2+^ signaling to regulate the hypoxia-induced response in *Arabidopsis*. Mass spectrometry-based phosphoproteomics exposed hypoxia-induced phosphorylation in seven different CDPKs (CPK1, 3, 6, 9, 12, 13 and 28), however, the abundance analysis established the substantial accumulation of CPK12. Utilizing Phos-tag immunoblot of CPK12 overexpression (OE) lines, the authors confirmed the accumulation of phosphorylated CPK12 in response to hypoxia. Further, reduced CPK12 phosphorylation during EGTA (ethylene glycol-bis(b-aminoethyl ether)-N,N,N’,N’-tetraacetic acid) treatment, assured Ca^2+^-dependent CPK12 phosphorylation. The site-directed mutagenesis of S (serine)-residue into nonphosphorylated A (alanine)-residue or phosphomimic residue D (aspartic acid), and subsequent mobility shift immuno-blot assays of transfected *Arabidopsis* protoplasts, revealed S186 as the phosphorylation site of CPK12. This site is conserved among all the seven CPKs phosphorylated during hypoxia. The *in-vitro* kinase assay of recombinant CPK12 and CPK12^S186A^ mutant proteins purified from *E. coli* further depicted S186 as a phosphorylation site.

The authors subsequently generated downregulated (RNAi) and OE transgenic lines, and performed phenotypic analyses to confirm the significance of phosphorylated CPK12 in hypoxia signaling. The enhanced tolerance of OE lines, with the higher number of photosynthetic leaves and cotyledons, chlorophyll content and dry biomass, and hypersensitivity of RNAi lines compared to WT during hypoxia treatment, assured vital function of CPK12 during hypoxia response in *Arabidopsis*. To establish the significance of S186 phosphorylation in CPK12 during hypoxia, Fan *et al*. also generated OE lines for mutants (CPK12^S186A^ and CPK12^S186D^) and used for phenotypic characterization.^10^ The CPK12^S186D^ OE lines showed enhanced tolerance as observed in the case of CPK12-OE lines, while CPK12^S186A^ OE lines displayed susceptibility toward hypoxia. These results demonstrated the importance of S186 phosphorylation in CPK12-mediated hypoxia signaling.

## CPK12 phosphorylates and stabilizes ERF-VII transcription factors during hypoxia signaling

The ERF-VII family proteins are earlier reported as crucial regulators during hypoxia signaling in *Arabidopsis*. A few of them such as RAP2.12, 2.2, and 2.3 function as activators for hypoxia-inducible genes.^13^ Therefore, the authors next established interactions between CPK12 and ERF-VII proteins (RAP2.12 and 2.3) using bimolecular fluorescence complementation (BiFC) and *in-planta* co-immunoprecipitation (co-IP) assays. These interactions were further confirmed *in-vitro* by pull-down assays using recombinant proteins. Moreover, it is worth noting that no other hypoxia-responsive CPK members demonstrated interaction with ERF-VII family proteins. The results suggest a specific interaction of CPK12 with these proteins.

The specific phosphorylation of ERF-VIIs by CPK12 has been ensured by *in-vitro* kinase assays. The authors then identified a conserved R-X-X-S/T motif in the N-terminal region of RAP2.12, 2.2, and 2.3, where threonine (T)-residue acts as a potential phosphorylation site. The *in-vitro* kinase assays using recombinant CPK12-mediated phosphorylation of RAP2.12 and 2.3, and their mutants (RAP2.12^T20A^ and RAP2.3^T23A^) established T-residue as a specific phosphorylation site. The *in-planta* co-transfection and immunoblotting revealed considerable accumulation of RAP2.12, 2.3, and 2.2 in the presence of CPK12, which suggests increased stability of ERF-VII proteins after CPK12-mediated phosphorylation. The significance of phosphorylation of ERF-VIIs during hypoxia is confirmed by generating OE lines for RAP2.12 and 2.3, and their mutants. Higher tolerance with more chlorophyll content in RAP2.12 and 2.3 OE lines established that the phosphorylation of the T20- and T23-residues in RAP2.12 and 2.3 is vital for hypoxia response in *Arabidopsis*. In addition, the authors used various combinations of co-expressing lines and established that the ERF-VIIs function downstream of CPK12.

The ERF-VII proteins are usually localized at the plasma membrane. Zhou *et al*.^3^ earlier reported that their stabilization and nuclear translocation during hypoxia are regulated by acyl-CoA binding proteins (ACBP1 and 2) and fatty acid desaturase (FAD3) through unsaturated acyl-CoA. The current study by Fan *et al*.^10^ also endorsed the above finding and established the significance of ACBP1/2- and FAD3-mediated acyl-CoA signal during CPK12-guided hypoxia responses in *Arabidopsis*.

## Nuclear translocation of CPK12 during hypoxia

The authors analyzed the nuclear localization of CPK12-GFP protein during hypoxia treatment using confocal microscopy. Since CPK12 lacks a nuclear localization signal (NLS), the study revealed phosphatidic acid (PA)-mediated nuclear translocation of CPK12, which is known for this function.^14^ Further, Zhou *et al*.^4^ earlier reported the role of PA in hypoxia signaling. The authors showed that arginine (R121)-residue at the KR-R motif is critical for PA binding to CPK12 by *in-vitro* and *in-planta* interaction studies. Inhibition of hypoxia-induced nuclear translocation of CPK12-GFP in the presence of 5-fluoro-2-indolyl des-chlorohalopemide (FIPI), an inhibitor for PA biosynthesis, reassured PA-mediated translocation of CPK12. On the contrary, 14–3-3k is identified as a CPK12 interacting protein that negatively regulates their nuclear translocation.^15^

## Hypoxia and ROS response

Hypoxia-induced increase in H_2_O_2_ in the plant tissues has been reported in earlier studies^7,16^ Wu *et al*.^7^ reported a time-dependent increase in H_2_O_2_ accumulation in the roots of *Arabidopsis*. However, the source of H_2_O_2_ generation is not clarified whether it is produced intracellularly in the cell organelles or extracellularly in the apoplast. The extracellular ROS production in the apoplast via respiratory burst oxidase homologs (RBOHs) is usually reported as the main source during environmental stress conditions,^17,18^ and the ROS signal amplifies through the ‘Ca-ROS hub’.^19^ Recently, a leucine-rich-repeat receptor kinase, HPCA1 (hydrogen-peroxide-induced Ca^2+^ increases 1) has been established as an extracellular sensor for the detection of H_2_O_2_ in the apoplast during stress conditions (Figure 1). This interaction leads to the activation of HPCA1 by autophosphorylation that ultimately stimulates the Ca^2+^ channels for Ca^2+^ influx,^11,12^ which might lead to the ‘Ca-ROS hub’ and ROS wave for the systemic response. Nevertheless, the association of HPCA1 with hypoxia signaling needs to be specifically validated in future research.

## Concluding remarks and future perspectives

In summary, the study by Fan *et al*.^10^ established the role of CPK12 as a sensor protein for decoding hypoxia-induced Ca^2+^ signature in *Arabidopsis* and transmitting the signal from cytoplasm-to-nucleus through phosphorylation cascade for hypoxia sensing (Figure 1). The study reveals the mechanism of Ca^2+^-induced autophosphorylation and PA-mediated translocation of CPK12 from cytoplasm-to-nucleus, where it phosphorylates and facilitates the accumulation of ERF-VII family proteins that ultimately activate the hypoxia-responsive genes. This study adds novel insights into the mechanism of Ca^2+^-mediated hypoxia signaling. However, a few queries remain unanswered. For instance, (i) how hypoxia-specific Ca^2+^ signature is generated in the cytoplasm and which Ca^2+^ channel specifically facilitates Ca^2+^ influx? Which component distinguishes the hypoxia-specific Ca^2+^ signature; CPK12 or Ca^2+^ channel? (ii) What is the activation mechanism of the Ca^2+^ channel during hypoxia? Is it directly induced by H_2_O_2_ or other ROS as proposed in an earlier study.^7^? Moreover, that study neither reported the source of H_2_O_2_ accumulation nor identified the hypoxia-specific Ca^2+^ channel. The H_2_O_2_-mediated activation of HPCA1 to elicit Ca^2+^ influx during numerous stress conditions^11,12^ indicates their plausible role in hypoxia, which might be interesting to explore in future studies.

## Data Availability

All the data are in the manuscript.
